# Ultrashort Vertical‐Channel van der Waals Semiconductor Transistors

**DOI:** 10.1002/advs.201902964

**Published:** 2019-12-23

**Authors:** Jinbao Jiang, Manh‐Ha Doan, Linfeng Sun, Hyun Kim, Hua Yu, Min‐Kyu Joo, Sang Hyun Park, Heejun Yang, Dinh Loc Duong, Young Hee Lee

**Affiliations:** ^1^ Center for Integrated Nanostructure Physics (CINAP) Institute for Basic Science (IBS) Suwon 16419 Republic of Korea; ^2^ Department of Energy Science Sungkyunkwan University Suwon 16419 Republic of Korea; ^3^ Department of Applied Physics Sookmyung Women's University Seoul 04310 Republic of Korea; ^4^ Department of Physics Sungkyunkwan University Suwon 16419 Republic of Korea

**Keywords:** 2D nanoelectronics, ultrashort channel, van der Waals semiconductors, vertical type transistors

## Abstract

Atomically thin 2D van der Waals semiconductors are promising candidates for next‐generation nanoscale field‐effect transistors (FETs). Although large‐area 2D van der Waals materials have been successfully synthesized, such nanometer‐length‐scale devices have not been well demonstrated in 2D van der Waals semiconductors. Here, controllable nanometer‐scale transistors with a channel length of ≈10 nm are fabricated via vertical channels by squeezing an ultrathin insulating spacer between the out‐of‐plane source and drain electrodes, and the feasibility of high‐density and large‐scale fabrication is demonstrated. A large on‐current density of ≈70 µA µm^−1^ nm^−1^ at a source–drain voltage of 0.5 V and a high on/off ratio of ≈10^7^–10^9^ are obtained in ultrashort 2D vertical channel FETs with monolayer MoS_2_ synthesized through chemical vapor deposition. The work provides a promising route toward the complementary metal–oxide–semiconductor‐compatible fabrication of wafer‐scale 2D van der Waals transistors with high‐density integration.

## Graphical abstract

Ultrashort vertical‐channel 2D van der Waals semiconductor transistors are fabricated with a controllable channel length of ≈10 nm via 2D vertical channels by squeezing an ultrathin insulating spacer between the out‐of‐plane source and drain electrodes, showing high performance with large on‐current density and high on/off ratio, together with the feasibility of large‐scale and high‐density fabrication.

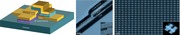

Ultrathin channels are required for scaling down nanodevices to overcome the short channel effects. Such a thin‐channel requirement cannot continue to be satisfied when using silicon‐based materials owing to the inevitable native surface states.^1^, ^2^ 2D van der Waals semiconductors with atomic‐scale thickness and free of surface dangling bonds are emerging as promising candidates to shrink the size of nanodevices further.^3^, ^4^, ^5^, ^6^, ^7^ 2D semiconductor transistors with gate lengths of even 1 nm have been demonstrated with gate electrodes made from single‐walled carbon nanotubes,^8^ which verifies the theoretical prediction of the advantage of atomically thin semiconductors when the channel length becomes ultrashort.^9^

2D nanotransistors with a short channel have been demonstrated through numerous approaches such as electron beam lithography (EBL),^10^, ^11^, ^12^, ^13^ shadow mask effect,^14^ self‐aligned nanowire gate,^15^, ^16^, ^17^ local phase transition,^18^ nanocracks,^19^ nanogrooves,^20^ and graphene nanogaps.^21^, ^22^, ^23^ However, such approaches are usually limited to a small scale or even a single device, rendering scale‐up difficult. Considering further practical applications, complementary metal–oxide–semiconductor (CMOS)‐compatible and well‐controllable fabrication processes for high‐density 2D nanotransistors in a large scale are still lacking. A vertically aligned transistor is another approach to achieve nanoscale devices for high integration density,^24^ and it has been demonstrated with quantum dots,^25^ nanowires or nanotubes,^26^, ^27^, ^28^ and thin films^29^, ^30^, ^31^, ^32^ as channel materials. However, the large‐scale alignment for 0D and 1D nanomaterials is still challenged. In the case of thin films, the thickness of the channel is still in the range of tens of nanometers, which cannot overcome the limitations of the short channel effect. In contrast, 2D van der Waals semiconductors with atomic‐scale thickness and free of dangling bonds could optimally match superior nanoelectronics.

Here, we report 2D vertical‐channel field‐effect transistors (2DVFETs) with a channel length of ≈10 nm by transferring various 2D van der Waals materials onto prefabricated out‐of‐plane aligned source‐insulating spacer‐drain (SID) patterns. The 2DVFETs with monolayer MoS_2_ grown through chemical vapor deposition (CVD) were obtained with an on‐current density over 20 µA µm^−1^ (70 µA µm^−1^ nm^−1^) at a source–drain voltage (*V*_ds_) of 0.5 V and a high on/off current ratio of ≈10^7^–10^9^. Similarly, high performance was achieved for mechanically exfoliated MoTe_2_ with an on‐current density ≈25 µA µm^−1^ at the same *V*_ds_ (over 100 µA µm^−1^ at *V*_ds_ = 2 V) and on/off ratio over 10^5^. In contrast to conventional planar FETs, 2DVFETs are composed of a vertically aligned source, 2D channel and drain, in which the channel length can be controllably scaled down to several nanometers via implementing the thickness of the insulating spacer between the bottom and top electrodes.

**Figure** **1**a,b illustrates our device structure and cross‐sectional transmission electron microscope (TEM) for one typical device. The key approach is to adopt a vertical configuration by transferring 2D semiconductors onto the prefabricated SID patterns with self‐aligned etching or continuous deposition. The vertical device is formed at the edge of the SID patterns, where ideally the channel length can be defined and controlled by tuning the thickness of the insulating spacer. The gate insulator is subsequently transferred or deposited with a following top‐side gate electrode. For demonstration, transferred layered hexagonal boron nitride (hBN) and atomic layer deposited Al_2_O_3_ were used as the insulating spacer and top‐side gate insulator. Monolayer MoS_2_ was used as the channel material with Au as the contact electrodes (details of the fabrication processes of 2DVFETs provided in Figure S1 in the Supporting Information). The cross‐sectional TEM image clearly shows the real structure of device, which reveals a continuous channel with well‐defined gate thickness. It is consistent with our proposal. We further investigated the existence of MoS_2_ channel after the transfer process and prior to top‐side gate fabrication by optical microscopy (Figure 1c‐I), top‐view and tilt‐view scanning electron microscope (SEM) images (Figure 1c‐II,III), and atomic force microscope (AFM) image (Figure 1c‐IV) of 2DVFET. All data clearly manifest that the 2D semiconductor channel attaches well with the SID pattern across the step.

**Figure 1:**
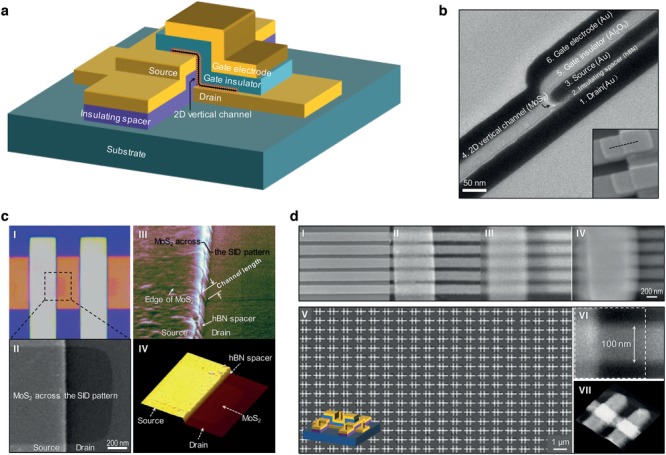
Schematic illustration of 2DVFETs and the feasibility of high‐density and large‐scale fabrication. a) Schematic illustration of the 3D view of the device layout, with mainly six layers, namely, the bottom electrode, insulating spacer, top electrode, 2D vertical channel, top‐side gate insulator, and gate electrode. b) Cross‐sectional TEM image of a typical 2DVFET. Inset: tilt‐view SEM image of the devices, where the black dashed curve marks the direction for the TEM image. c) Confirmation of continuous MoS_2_ monolayer well‐attaching to the SID patterns. I) Top‐view optical image. II) Top‐view SEM image. III) Tilt‐view SEM image. IV) Tilt‐view AFM image. d) Demonstration for high‐density and large‐scale fabrication. I–IV) Top‐view SEM images showing the key fabrication processes of an array of 2DVFETs with the bottom electrode width of ≈200 nm. V) Top‐view SEM image of the fabricated large‐scale high‐density 2DVFETs with CVD‐grown MoS_2_ prior to top‐side gate fabrication and metal interconnection. Inset: schematic illustration of a single CMOS of 2DVFETs. VI) Higher‐magnification SEM image of a single 2DVFET in (d‐V) with the electrode width of ≈100 nm and transferred MoS_2_ shown in the dotted box as the channel. VII) AFM image of the devices in (d‐V)

To demonstrate the feasibility of large‐scale and high‐density fabrication, we fabricated an array of 2DVFETs with bottom electrodes of width ≈200 nm with four key fabrication processes in Figure 1d‐I–IV (see also Figure S2a in the Supporting Information). The channel length is determined by the spacer. The device size is almost dominated by the width of the electrodes. Furthermore, Figure 1d‐V–VII shows the fabricated large‐scale and high‐density integration of 2DVFETs with CVD‐grown MoS_2_ prior to top‐gate fabrication and metal interconnection (see also Figure S2b in the Supporting Information). In our structure, the average size of the single cell is ≈10^2^ × 10^2^ nm^2^. The wafer‐scale ultrashort 2DVFETs could be viable by fabricating large‐scale SID‐like patterns using photolithography,^25^ together with the currently available 2D semiconductor growth^33^, ^34^ and transfer technology.^35^

To evaluate the potential performance of 2DVFETs, we demonstrate here the characteristics of devices without several etching processes which normally degrade the device performance,^36^ and relatively thick hBN was used as gate insulator instead of Al_2_O_3_ as demonstrated in the TEM image (Figure 1b) to avoid any gate leakage. The performance of a representative 2DVFET with CVD‐grown monolayer MoS_2_ is demonstrated in **Figure** **2**. Figure 2a illustrates the cross‐section of the device, with Au as source (S), drain (D), and gate (G) electrode material, monolayer MoS_2_ as channel material, and exfoliated hBN as both spacer and gate insulator. In this device, the thickness of the hBN insulating spacer was ≈12.6 nm, which was supposed to determine the channel length, and the channel width was ≈8.1 µm. (More details of the device provided in Figures S3 and S4 in the Supporting Information.)

**Figure 2:**
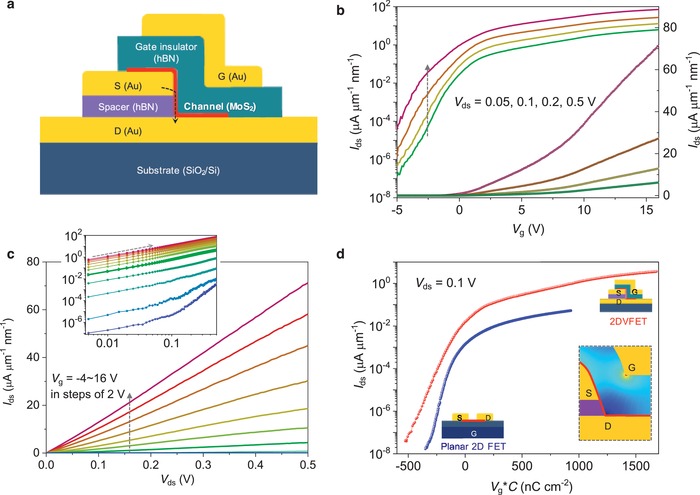
Electrical characteristics of the ultrashort CVD‐grown MoS_2_‐based 2DVFETs. a) Cross‐sectional schematic illustration of the device. b) *I*_ds_–*V*_g_ transfer characteristics of the MoS_2_‐based 2DVFET in the linear form and logarithm, with *V*_ds_ = 0.05, 0.1, 0.2, and 0.5 V, and *V*_g_ = −5 to 16 V. c) *I*_ds_–*V*_ds_ output characteristics of the device. Inset: *I*_ds_–*V*_ds_ output curves at log–log scale. d) Comparison of device performance between 2DVFET and conventional planar 2D FET with CVD‐grown monolayer MoS_2_. Here, we take *V*_g_ × *C* as the *X*‐axis to consider the influence of thickness and dielectric constant differences of hBN and SiO_2_ as the gate insulators in 2DVFETs and planar devices, respectively. Inset: schematic illustration of the local enhancement of electric field in 2DVFETs

The transfer curves are provided in Figure 2b by sweeping the top‐gate voltage (*V*_g_), with *V*_ds_ across the vertical short channel held at different fixed values. When the *V*_g_ varies from 16 to −5 V, the vertical MoS_2_ channel switches from the “on” state to the “off” state, with an on/off current ratio over 10^7^ for *V*_ds_ = 0.05, 0.1, and 0.2 V, and ≈2 × 10^6^ for *V*_ds_ = 0.5 V, which are similar to that of reported long‐channel devices. The on current density at *V*_ds_ = 0.5 V and *V*_g_ = 16 V can reach 21.5 µA µm^−1^, which is among the best performance of monolayer MoS_2_‐based short channel transistors at similar *V*_ds_ and gate modulation,^15^, ^16^, ^18^, ^19^, ^21^, ^23^ and much higher than that of conventional long‐channel devices with MoS_2_.^3^, ^37^ The subthreshold swing (SS) of this device is ≈0.7 V dec^−1^, which can be reduced further with a thinner gate insulator and cleaner interface.^38^ We expect that the value can be reduced to 77 mV dec^−1^ if equivalent oxide thickness of gate is reduced to 0.9 nm as high‐*k* metal gate^39^ in silicon technology following the equation SS*_t_*_′_ = SS_0_ + (SS*_t_* – SS_0_) × (*t*′/*t*) × (*ε_t_*/*ε_t_*_′_), where *t* is the thickness of the gate insulator, SS_0_ is the thermionic limitation of 60 mV dec^−1^ at room temperature for conventional FETs, and ε is the dielectric constant of the gate insulator.^40^ For a relatively fair comparison with silicon‐based devices, we calculated the on‐current density divided by the thickness. The value is ≈70 µA µm^−1^ nm^−1^ for the monolayer MoS_2_‐based 2DVFET at *V*_ds_ = 0.5 V, which is comparable to ≈56 µA µm^−1^ nm^−1^ at *V*_DD_ = 0.5 V (≈130 µA µm^−1^ nm^−1^ at *V*_DD_ = 0.7 V) for the silicon fin field‐effect transistor (FinFET) at Intel 14 nm node^41^ and ≈108 µA µm^−1^ nm^−1^ at *V*_DD_ = 0.5 V (≈250 µA µm^−1^ nm^−1^ at *V*_DD_ = 0.7 V) for FinFET at 10 nm node.^42^ (See Table S1 in the Supporting Information for a comprehensive comparison between 2DVFET and previous reported short channel 2D semiconductor transistors, and Table S2 in the Supporting Information for a comparison between 2DVFET and Si‐based FET.) The current leakages of both the hBN spacer and top gate are negligible (Figure S5, Supporting Information). The full passivation of the channel part with the top gate guarantees the tenable stability of the device performance for 2DVFETs (Figure S6, Supporting Information).

The output curves in Figure 2c show a near‐linear relation with a slight S‐shape between the output current (*I*_ds_) and *V*_ds_, particularly at the on‐state of the device. The log–log scale curves of *I*_ds_–*V*_ds_ manifest more apparently the near‐linearity at *V*_g_ = 0–16 V, with slopes close to 1. Such a near‐linearity suggests that the contact is relatively close to Ohmic contact at the on‐state. In contrast, near the off‐state, the slope of the log–log scale curve is completely nonlinear at *V*_g_ = −2 to −4 V, which indicates a non‐Ohmic contact. However, the slight S‐shape of the output curves at the on‐state indicates that the contact is not ideal, which will be discussed quantitatively later.

Figure 2d shows a schematic comparison of device performance between 2DVFET and conventional planar 2D FET. The transport curves of the CVD‐grown monolayer MoS_2_‐based 2DVFET and typical planar 2D FET with micrometer‐scale channel are presented. It is evident that 2DVFETs show much higher on‐current by almost two orders of magnitude than micrometer‐sized planar 2D FETs, whereas high on/off ratios are retained. In our 2DVFETs, the gating electric field is not uniform and would be strongest around the corner (illustrated in the inset of Figure 2d) due to the nonplanar curvature of the device structure. However, such local enhancement of gating effect around the corner would be advantageous for turning on and off the channel more effectively (see Figure S7 in the Supporting Information for more detailed discussion).

To understand the device performance and the nature of the contact in 2DVFETs (Figure S8, Supporting Information),^43^, ^44^, ^45^ we performed temperature‐dependent *I*_ds_–*V*_ds_ measurements (**Figure** **3**a left), and further calculated the Schottky barrier with a 2D thermionic emission model, which is suitable for normal 2D transistors,^46^, ^47^ *I*_ds_ =  *AT* ^3/2^exp(−*qϕ*_B_/*k*_B_ *T*)[exp (*qV*_ds_/*nk*_B_ *T*) − 1], where *I*_ds_ is the current, *A* is Richardson's constant, *T* is the temperature, *q* is the charge of electron, ϕ_B_ is the barrier between the semiconductor channel and metal contact, *k*_B_ is the Boltzmann constant, and *n* is an ideality factor of the Schottky diode. When *V*_ds_ ≥ 3*k*_B_ *T*/*q*, exp (*qV*_ds_/*nk*_B_ *T*) ≫ 1, $I_{\mathrm{ds}}\;\approx \;AT^{3/2}\exp(-q\phi_{B}/k_{B}T+qV_{\mathrm{ds}}/nk_{B}T)$, and $\ln(I_{\mathrm{ds}}/T^{3/2})\;\approx -q(\phi_{B}-V_{\mathrm{ds}}/n)/k_{B}T+\ln(A)$. From the temperature‐dependent *I*_ds_–*V*_ds_ curves, we can observe that the on‐current with two‐probe measurement at low temperature (down to 30 K) is below one order of magnitude smaller than that at room temperature (300 K) (less than 50% reduction at *V*_ds_ of 1 V), indicating a good contact in 2DVFETs. Arrhenius plots of ln(*I*_ds_/*T* ^3/2^) ∼1000/*T* for various values of *V*_ds_ bias are calculated to evaluate the detailed ϕ_B_ (Figure 3a right). The slope values of Arrhenius plots (*S*_A_) can be subsequently plotted as a function of *V*_ds_. We can subsequently calculate ϕ_B_ from the *y*‐intercepts (*S*_0_) of *S*_A_–*V*_ds_ curves according to *S*_0_ = −*qϕ*_B_/(1000*k*_B_).

**Figure 3:**
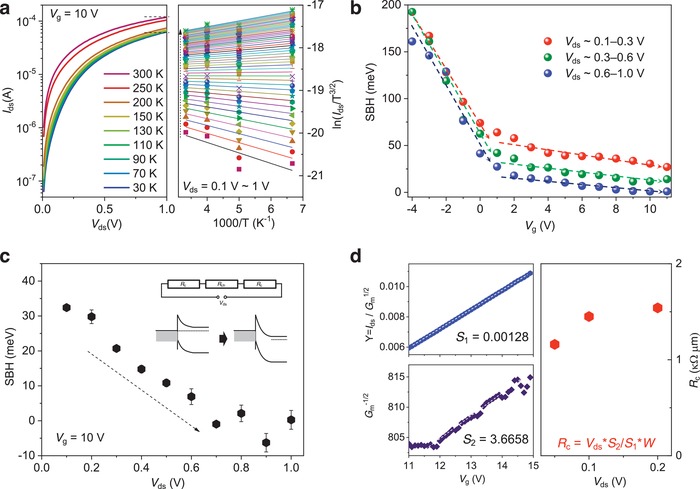
Schottky barrier height and contact resistance calculation for the MoS_2_‐based 2DVFETs. a) Typical temperature‐dependent *I*_ds_–*V*_ds_ curves at *V*_g_ = 10 V (left). The corresponding Arrhenius plots of ln(*I*_d_/*T* ^3/2^)∼1000/*T* (right). b) Calculated SBH depending on *V*_g_. Here, we take three separated near‐linear ranges (0.1–0.3, 0.3–0.6, and 0.6–1.0 V) to calculate the SBH. c) Calculated detailed SBH changing with *V*_ds_ at *V*_g_ = 10 V. Inset: circuit diagram involving both contact resistance and channel resistance for the device and schematic illustration to show tilt of the band bending close to the barrier. d) Typical processes of the *Y*‐function method for contact resistance calculation (left) and the calculated contact resistance at *V*_ds_ = 0.05, 0.1, and 0.2 V (right). Here, *G*_m_ is the transconductance calculated from the transfer curves

Owing to the anomalous nonlinearity of *S*_A_–*V*_ds_ curves (Figure S9, Supporting Information), we extracted the Schottky barrier height (SBH) with different ranges of *V*_ds_. The calculated SBH from three separated ranges of *V*_ds_ changing with *V*_g_ is shown in Figure 3b. The barrier calculated at the *V*_ds_ range 0.6–1.0 V is reduced from 200 meV to below 10 meV as *V*_g_ increases. Interestingly, the calculated barrier heights are strongly varied with *V*_ds_ at *V*_g_ larger than 0 V, namely, close to on‐state. To manifest this effect, the variation of SBH with *V*_ds_ at *V*_g_ of 10 V is shown in Figure 3c. Such a drain‐induced Schottky barrier lowering effect in our 2DVFETs originates from the tilting of the band at the contact junction, resulting in reduction of the Schottky barrier width and consequently a possible tunneling current through the Schottky barrier.^22^ This phenomenon is predominant when the resistance of the channel becomes small due to its nanometer scale length at on‐state, which gives rise to a large proportion of source–drain bias dropped on the contact part. We note that such an anomalous effect does not occur in long‐channel devices,^46^ indicating the crucial short‐channel evidence of our devices. In contrast, at the off‐state, the channel resistance is not sufficiently low to observe this drain‐induced Schottky barrier lowering effect. This drain‐induced Schottky barrier lowering effect discussed above occurs at the on‐state and is not evident at the off‐state, which helps improve the on‐current while not influencing the on–off ratio appreciably.

We also used the *Y*‐function method^48^, ^49^ to evaluate the contact resistance of the MoS_2_ 2DVFETs. The contact resistance at the on‐state is ≈1.2–1.5 kΩ µm (Figure 3d; Figure S10, Supporting Information), which is comparable to that of a strategy such as hBN/Ni^50^ and hBN/Co^51^ for contact improvement. For comparison, the contact resistance for long‐channel devices with the channel transferred onto the prepatterned electrodes is over 30 kΩ µm (Figure S10, Supporting Information), which is similar to that of Ti/Au with an annealing strategy.^52^ This again confirms the reasonable contact in 2DVFETs. It is noted that the saturation of the drain current can be observed in lateral devices with the contact resistance of ≈1.3 kΩ µm (comparable to our device of 1.2–1.5 kΩ µm).^53^ Therefore, we believe that the nonsaturation of the drain current (Figure 2c) in our devices origins from the drain‐induced Schottky barrier lowering effect, which is also the sign of a short channel.

The contact is still not ideal compared to the contact resistance of the high‐performance Si transistors (less than 0.1 kΩ µm) (as presented in Table S2 in the Supporting Information). This is still a challenge in 2D semiconductor electronics. To improve the contact for 2DVFETs further, a better work‐function‐matched metal could be selected,^44^ together with better deposition condition^54^ and proper annealing treatment.^55^ Graphene could also be taken into account as a good contact for transition metal dichalcogenides (TMDs) (Figure S11, Supporting Information).^47^

To confirm the generality of our method, we also fabricated ultrashort 2DVFETs with mechanically exfoliated MoTe_2_. **Figure** **4** shows the device structure and performance with transfer characteristics and output characteristics. Few‐layered hexagonal MoTe_2_ (thickness ≈3.7 nm) was exfoliated and transferred onto the SID pattern as a vertical channel (Figure 4a). In this MoTe_2_‐based 2DVFET, the thickness of the hBN insulating spacer is ≈16.5 nm, and hBN gate insulator ≈30 nm (Figure S4, Supporting Information). The on‐current density achieves ≈25 µA µm^−1^ at *V*_ds_ of 0.5 V (over 100 µA µm^−1^ at *V*_ds_ = 2 V), which is the highest performance for MoTe_2_ field effect transistors, to the best of our knowledge. The on/off ratio keeps over 3 × 10^5^ for *V*_ds_ = 0.1, 0.5, and 1 V (Figure 4b). The SS value is ≈290 mV dec^−1^, much better than previous reports of long channel devices with consideration of the gate difference.^56^, ^57^ The output curves of the device in Figure 4c are similar to those of monolayer MoS_2_‐based 2DVFETs. The contact resistance calculated using the *Y*‐function method is less than 2 kΩ µm (Figure S9, Supporting Information). A comparison of the device performance between the MoTe_2_‐based 2DVFET and planar FET demonstrates the advantages of our strategy again (Figure 4d). To achieve higher on‐current, other 2D van der Waals semiconductors^5^, ^7^ or nanometer‐thin film^58^ with higher mobility could be good candidates with the same strategy.

**Figure 4:**
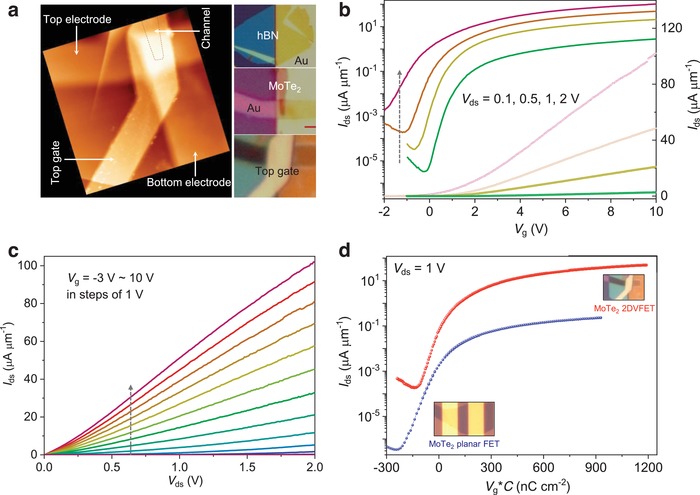
Mechanically exfoliated MoTe_2_‐based 2DVFET. a) AFM image (left) and optical image (right bottom) of the device, optical image of the transferred hBN spacer (right top) before top electrode fabrication, and optical image of MoTe_2_ transferred onto the fabricated SID pattern as the vertical channel (right middle). b) *I*_ds_–*V*_g_ transfer characteristics of the device in the linear form and logarithm, with *V*_ds_ = 0.1, 0.5, 1, and 2 V. c) *I*_ds_–*V*_ds_ output characteristics of the device with *V*_g_ = −3 to 10 V. d) Comparison of device performance between MoTe_2_‐based 2DVFET and conventional planar FET

Although good performance of 2DVFETs is demonstrated in our current configuration, there are several issues that should be further studied, especially the issues of the gate length and the SS value. Both of them are related to the optimization of the gate. To decrease the gate length in 2DVFETs, we propose that a short gate length can be achieved by the sidewall etching technique normally used in the spacer patterning in industry.^41^, ^59^ The SS value of the devices can be decreased by reducing the equivalent oxide thickness of the gate and improving the quality of the interface between the gate and channel.^38^ (See Table S3 in the Supporting Information for more detailed discussion.)

In conclusion, we have demonstrated a new type of 2D van der Waals semiconductor‐based ultrashort vertical channel FETs, 2DVFETs. By prepatterning an out‐of‐plane aligned SID structure as the contact and post‐transferring 2D semiconductors (MoS_2_, MoTe_2_) as channel materials, the channel length shrinks to ≈10 nm and the devices show high performance. Typically, the on‐current of CVD‐grown monolayer MoS_2_‐based 2DVFETs can reach over 70 µA µm^−1^ nm^−1^ at a *V*_ds_ of 0.5 V, and the on/off ratio approaches 10^7^ to 10^9^. The contact of 2DVFETs is nearly Ohmic at the on‐state. Moreover, the channel length of 2DVFETs can be simply controlled by the thickness of the atomically thin insulating spacer between the source and drain electrodes, which can be compatible with current CMOS technology in industry. With the development of 2D materials growth and transfer technologies together with the improvement of insulator materials, wafer‐scale high‐density 2DVFETs could be possible and may provide a promising route to pursue Moore's law further.

## Experimental Section

*Synthesis of MoS_2_ by CVD Technology*:^60^ Monolayer MoS_2_ was synthesized via two‐temperature‐zone atmospheric pressure chemical vapor deposition (APCVD) with ammonium heptamolybdate (AHM, Sigma‐Aldrich, 431346) as molybdenum (Mo) source and sulfur powder (S, Sigma‐Aldrich, 213292) as S source. A mixture of AHM solution (0.2 g AHM dissolved in 30 mL deionized (DI) water), NaOH solution (0.1 g NaOH dissolved in 30 mL DI water), and iodixanol solution (Sigma‐Aldrich, OptiPrep, D1556) was first prepared in the volume ratio of 0.3:3:0.5, followed by spin coating onto SiO_2_/Si wafer. Subsequently, the prepared SiO_2_/Si wafer with Mo source was placed in the high‐temperature zone (750 °C with heating‐up time of 6 min) of the APCVD system and reacted with S source placed in the low‐temperature zone (180 °C with the same heating‐up time) under a continuous gas flow of argon (500 sccm). The growth time was 9 min. Finally, the furnace was naturally cooled to room temperature.

*Preparation of MoTe_2_ and hBN Thin Flakes via Mechanical Exfoliation*: The layered materials were mechanically exfoliated with blue tape (Ultron systems, Inc.) to reduce the containment during exfoliation.

*Transfer Technology of 2D Flakes*: The polyvinyl alcohol (PVA)/poly(methyl methacrylate) (PMMA) method was used for transfer. For the transfer of exfoliated flakes, flakes were first exfoliated with a scotch tape onto the preprepared PVA/PMMA‐coated substrate. Appropriate flakes were observed using an optical microscope. The sample was subsequently dipped into hot water (80–90 °C), and the flake/PMMA layer was detached from wafer after minutes. Flakes were transferred onto a special holder with a hole first, and thereafter aligned and contacted with the target pattern under the optical microscope. The flake/PMMA layer was finally detached from the holder at 140 °C. After cooling, PMMA was removed with acetone. For CVD‐grown monolayer MoS_2_, similar processes were performed. In contrast to the flakes directly exfoliated on the PVA/PMMA‐coated substrate, the CVD‐grown monolayer MoS_2_ was coated on a Si/SiO_2_ substrate with PMMA, and the flake/PMMA was detached from the substrate with hydrofluoric acid. After washing with DI water for several times, the flake/PMMA was flipped up and down with plasma‐treated hydrophilic polyethylene terephthalate film or polydimethylsiloxane film, and transferred onto the holder. The following processes were the same.

*Device Fabrication*: For self‐aligned etching‐based fabrication, few‐layered hBN flakes (from 2D Semiconductors Co., Ltd.) were first exfoliated and transferred onto the prefabricated bottom electrodes, followed by EBL and thermal evaporation to fabricate the top electrodes. Au was deposited as the electrode material with a stable surface and good wetting to TMDs. Subsequently, with the top electrode as the mask, the part of unpassivated hBN was etched with sulfur hexafluoride (SF_6_) using reactive‐ion etching (RIE). TMD flakes were thereafter transferred onto the well‐fabricated SID patterns (and etched to defined size with RIE) as vertical channels. Finally, another hBN was transferred onto the vertical channel part as the top‐side gate insulator (or high‐K dielectric layer deposited with atomic layer deposition), followed by Au deposited as the gate electrode. For self‐aligned continuous deposition‐based fabrication, SID patterns were prepared with continuous deposition of spacer layer and top electrode, other processes were similar. (See also Figure S1 in the Supporting Information.)

*Device Characterization*: TEM, SEM, and AFM characterizations for the devices were performed with the JEOL JEM‐ARM200F, JEOL JSM‐6510, and NanoNavi SPA‐400SPM, respectively. The electrical characterization was carried out in vacuum (≈10^−6^ Torr) with a probe station system and semiconductor analyzer (Keithley 4200 system). Low‐temperature measurement was performed with a Lake Shore probe system CRX‐VF.

## Conflict of Interest

The authors declare no conflict of interest.

## Author Contributions

J.B.J., D.L.D., and Y.H.L. conceived and designed the project. J.B.J., M.‐H.D., and L.F.S. fabricated the devices and performed electrical characterizations. H.K. and H.Y. prepared the CVD samples. J.B.J. and M.‐K.J. performed the contact resistance calculation using the *Y*‐function method. J.B.J. and S.H.P. performed the SEM and TEM characterizations. H.Y. provided the Lake Shore system setups. J.B.J., D.L.D., and Y.H.L. analyzed the data and co‐wrote the manuscript. All authors discussed the results and reviewed the manuscript.

## Supporting information

Supporting Information
