# Supplementary material for: Ultrashort Vertical‐Channel van der Waals Semiconductor Transistors
Source: Adv Sci (Weinh). 2019 Dec 23;7(4):1902964. doi: 10.1002/advs.201902964 (PMC7029639; doi:10.1002/advs.201902964)
Supplement: Supplementary file 1 — Supporting Information [file ADVS-7-1902964-s001.pdf]

Copyright WILEY-VCH Verlag GmbH & Co. KGaA, 69469 Weinheim, Germany, 2019.

Supporting Information

for *Adv. Sci.,* DOI: 10.1002/advs.201902964

Ultrashort Vertical-Channel van der Waals Semiconductor Transistors

Jinbao Jiang, Manh-Ha Doan, Linfeng Sun, Hyun Kim, Hua Yu, Min-Kyu Joo, Sang Hyun Park, Heejun Yang, Dinh Loc Duong, and Young Hee Lee**

Copyright WILEY-VCH Verlag GmbH & Co. KGaA, 69469 Weinheim, Germany, 2019.

Supporting Information

Ultra-Short Vertical-Channel van der Waals Semiconductor Transistors

Jinbao Jiang, Manh-Ha Doan, Linfeng Sun, Hyun Kim, Hua Yu, Min-Kyu Joo, Sanghyun Park, Heejun Yang, Dinh Loc Duong, and Young Hee Lee**

J. B. Jiang, Dr. H. Kim, Dr. H. Yu, S. H. Park, Prof. H. J. Yang, Prof. D. L. Duong, Prof. Y. H. Lee
Center for Integrated Nanostructure Physics (CINAP)
Institute for Basic Science (IBS)
Suwon 16419, Republic of Korea
E-mail: ddloc@skku.edu; leeyoung@skku.edu
J. B. Jiang, Dr. M.-H. Doan, Dr. L. F. Sun, Dr. H. Kim, Prof. H. J. Yang, Prof. D. L. Duong, Prof. Y. H. Lee
Department of Energy Science
Sungkyunkwan University
Suwon 16419, Republic of Korea
Prof. M.-K. Joo
Department of Applied Physics
Sookmyung Women's University
Seoul 04310, Republic of Korea
Prof. Y. H. Lee
Department of Physics
Sungkyunkwan University
Suwon 16419, Republic of Korea

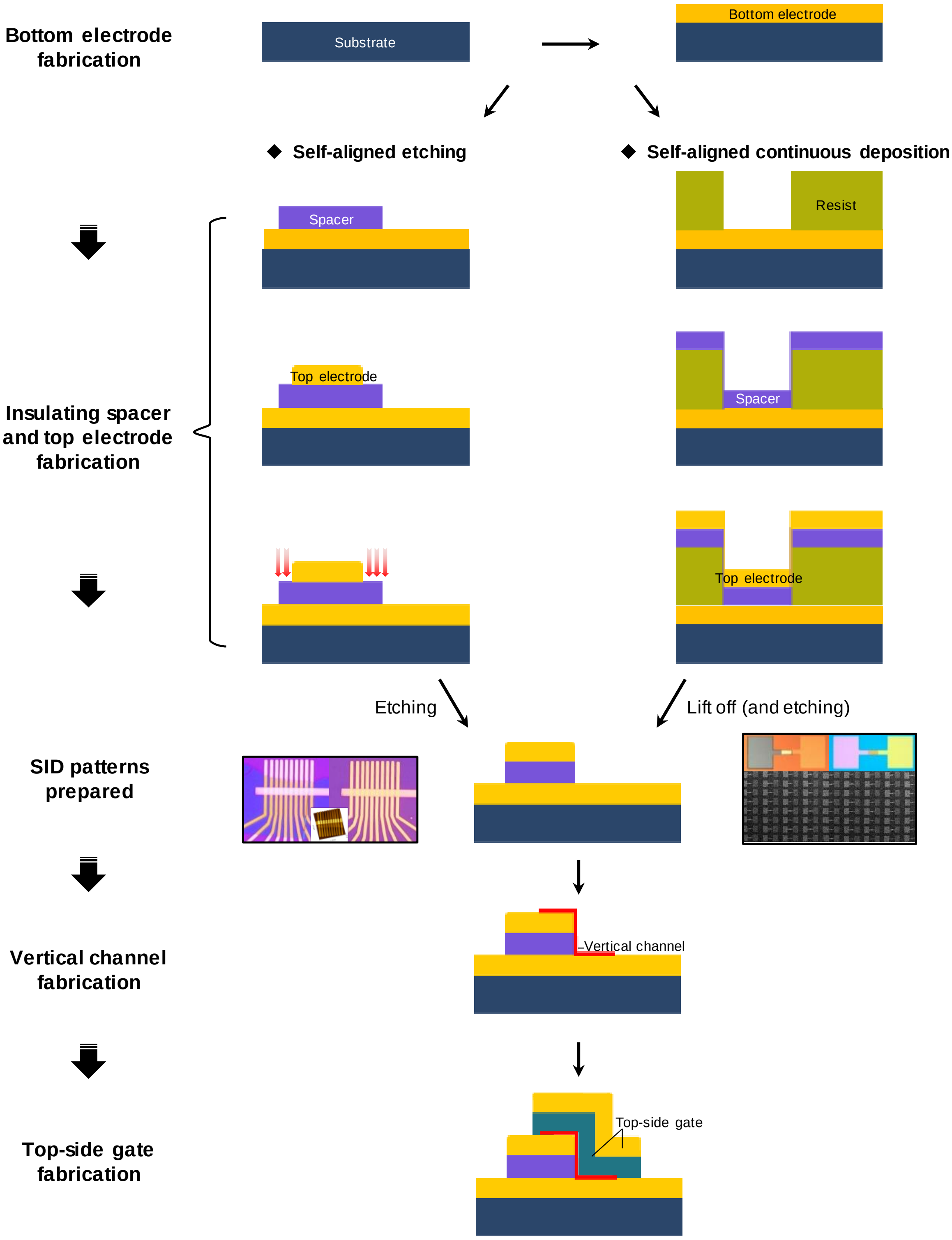

Figure S1. The main fabrication processes of 2DVFETs. For the fabrication of the source-insulating spacer-drain (SID) patterns, we could use the etching approach for hBN (self-aligned etching) or normal patterning deposition for oxides (self-aligned continuous deposition). Both layered hBN and deposited oxides could be used for the insulating spacer and gate insulator.

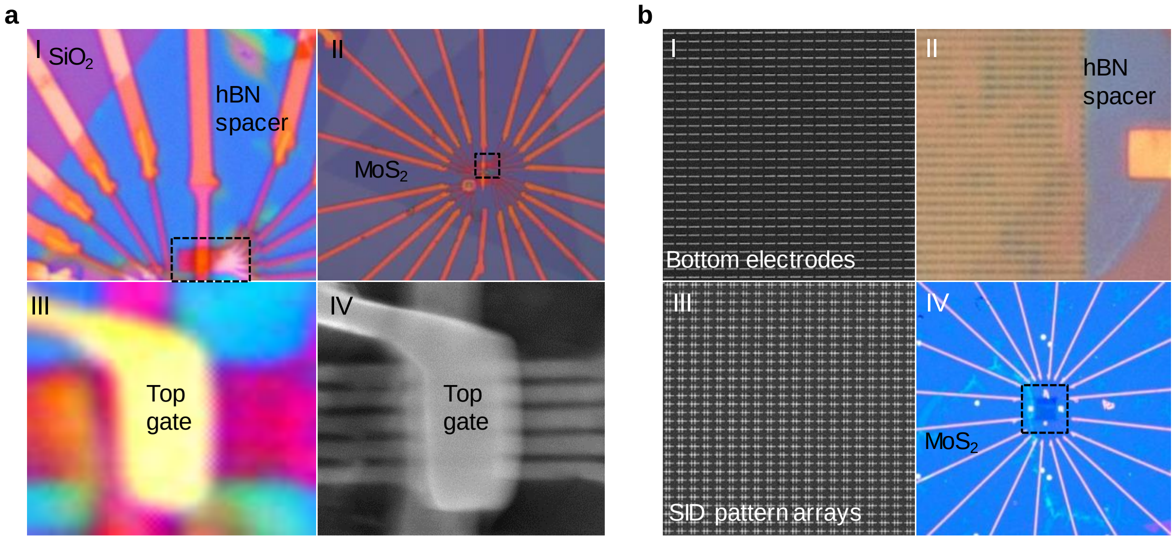

Figure S2. Supporting information for the high-density and large-scale fabrication of 2DVFETs. (**a-b**) Optical and SEM images of the key fabrication processes and detailed components of the high-density and large-scale 2DVFETs arrays in Figure 1d.

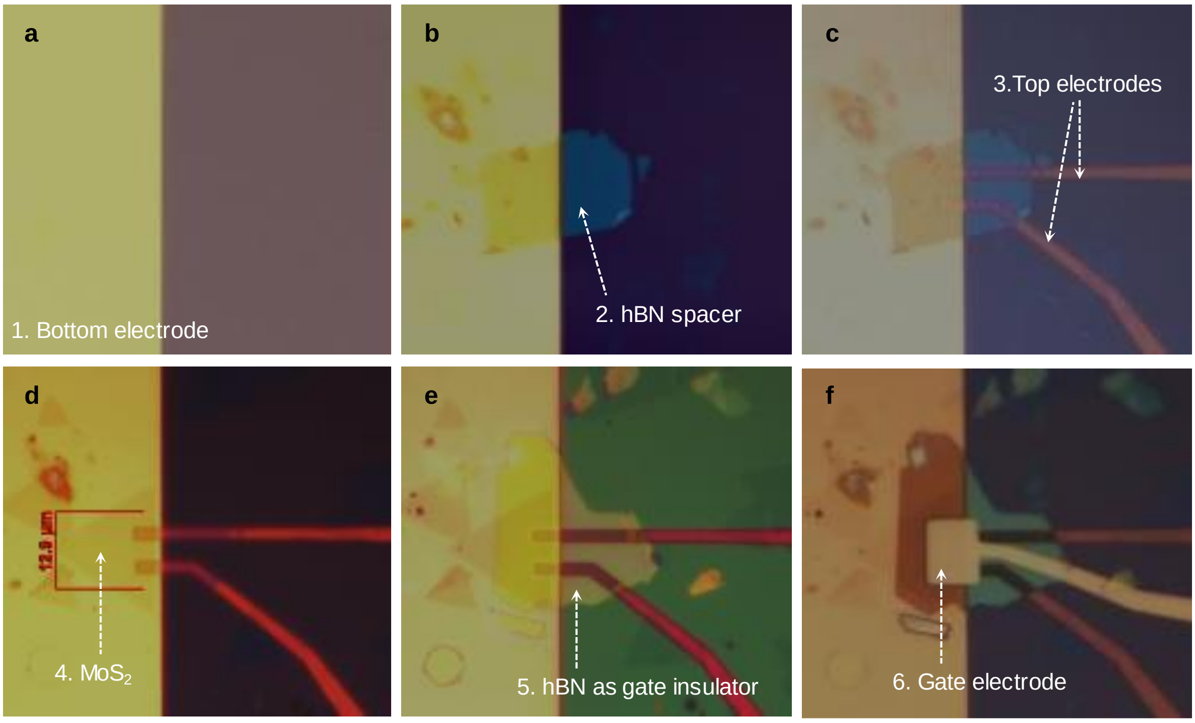

Figure S3. Optical images to show the main fabrication processes of CVD-MoS_2-based 2DVFETs. **(a)** Bottom electrodes. **(b)** Few-layer hBN as the insulating spacer on the bottom electrode. **(c)** Top electrode on the hBN spacer before etching the hBN spacer. **(d)** Monolayer CVD MoS_2 as the channel on the well-fabricated SID pattern after etching hBN spacer. **(e)** Another hBN as the top-gate insulator. **(f)** Top-gate electrodes. Here, two devices are fabricated simultaneously.

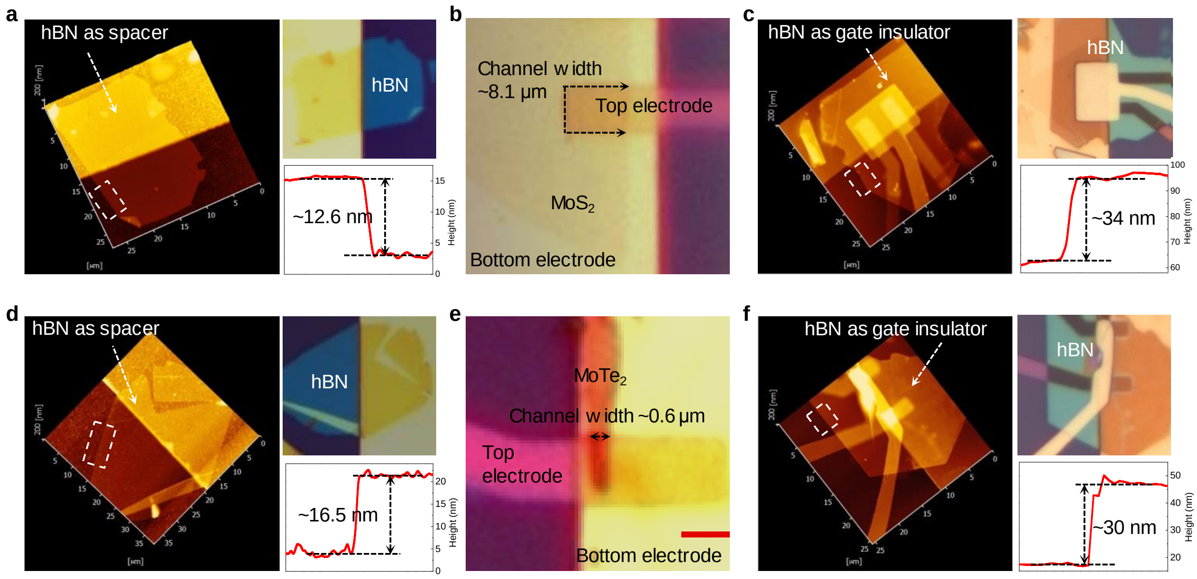

Figure S4. Detailed physical structure of the MoS_2- and $MoTe_2$-based 2DVFETs. For MoS_2 device (a-c). **(a)** 3D view AFM image of the hBN spacer before etching with the top-view optical micrograph (right top), and the line profile of the marked region in AFM, indicating thickness of 12.6 nm (right bottom). **(b)** Top-view optical image of MoS_2 on the SID pattern before gate fabrication. The total channel width is approximate 8.1 μm as marked by the black dashed curve. **(c)** 3D view AFM image of the final devices with the top-view optical image (two devices are fabricated). The height of the hBN top-gate insulator is ~34 nm. The same process for $MoTe_2$ device(d-f).

Table S1. Technical summary of typical short channel 2D semiconductor transistors.

		EBL				Self-aligned 1D gate			
**Reference**		CM Hu, et al.[1]	CM Hu, et al.[2]	CM Hu, et al.[3]	P. D. Ye, et al.[4]	Eric Pop, et al.[5]	K Banerjee, et al.[6]	Lei Liao, et al.[7]	Ali Javey, et al.[8]
**Schematic of device structure**		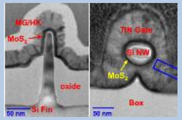	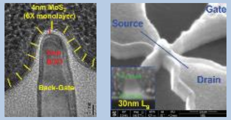	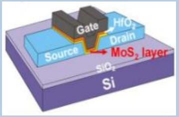	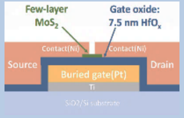	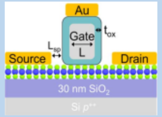	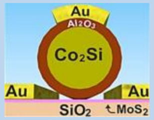	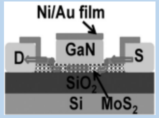	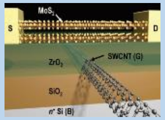
**Key strategy**		Hybrid Si/TMD 3DFETs & CVD insitu growth	TMD FinFET & CVD insitu growth	Si nano groove & CVD insitu growth	Chloride Doping & EBL patterning	Self-aligned NW gate	Self-aligned NW gate	Self-aligned NW gate	Self-aligned SCNT gate
**Device parameters and performance**	**Channel Materials**	CVD MoS_2 3~16 layers	CVD MoS_2 6 layers	CVD MoS_2 7 layers	Exfoliated MoS_2 6~10 layers	**CVD MoS_2 monolayer**	**CVD MoS_2 monolayer**	Exfoliated MoS_2 few layers	Exfoliated MoS_2 2 layers
	**Channel length**	50 nm	30 nm	10 nm	10 nm	~10 nm	~10 nm	~90 nm	~600 nm (Gate 1 nm)
	**Gate**	4 nm HfO_2 (EOT ~1.6 nm)	4 nm SiO_2 (EOT 4 nm)	5 nm HfO_2 (EOT ~2 nm)	7.5 nm HfO_x (EOT ~3 nm)	5 nm Al_2O_3 (EOT ~5 nm)	6 nm Al_2O_3 (EOT ~5 nm)	/	5 nm $Zr0_2$ (EOT ~0.8 nm)
	**V_g range**	0~2 V (V_{th} ~-0.5 V)	0~3 V (V_{th} ~-0.6 V)	2~-4 V P type	-2~2 V (V_{th} ~-0.8 V)	-5~0 V (V_{th} ~-3.8 V)	-2~2 V (V_{th}~-0.6 V)	-3~1 V (V_{th}~-1.6 V)	-3~0.5 V (V_{th}~-2 V)
	**I_{on} /layer number at V_{ds} = 0.5 V**	~ 114 μA/μm / (3~16)	~ 160 μA/μm / 6	~ 32 μA/μm / 7	~180 μA/μm / (6~10)	~80 μA/μm / 1	~6 μA/μm / 1	~35 μA/μm / >2	~7 μA/μm / 2
	**On/off ratio**	10^5	10^5	$>10^5$	10^6	$<10^3$	$\sim10^6$	$<10^6$	$\sim10^6$
	**SS**	~142 mV/dec	~143 mV/dec	~200 mV/dec	~200 mV/dec	250 mV/dec	180 mV/dec	150 mV/dec	65 mV/dec
**Contact**	**Contact Material**	/	W top contact	Doped poly-Si bottom contact	Ni top contact	Ultra-HV Ti-Au top contact	Au top contact	Ni/Au top contact	Ni top contact
	**R_c**	/	/	/	/	~1.7 KΩ μm	/	/	/
**Feasibility of large-scale and high-density fabrication**		Difficult	Difficult	Difficult	Difficult	Difficult	Difficult	Difficult	Difficult

Table S1 continued. Technical summary of typical short channel 2D semiconductor transistors.

		Nano gaps				Block copolymer		Shadow mask	Vertical structure
**Reference**		XF Duan,et al.[9]	Jun He, et al.[10]	A Nourbakhsh, et al.[11]	GY Zhang, et al.[12]	A Nourbakhsh, et al.[13]		Chuan Wang, et al.[14]	This work
**Schematic of device structure**		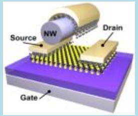	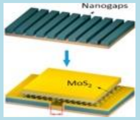	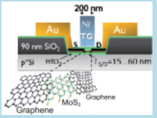	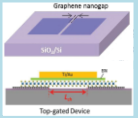	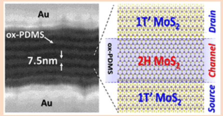		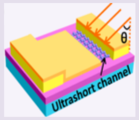	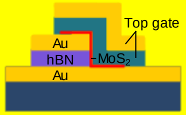
**Key strategy**		Nanogaps from Nanowire assisted lift-off	Nanogaps from corrosion cracking	Nanogaps from Gr EBL -assisted etching	Nanogaps from Gr-boundary etching	Block copolymer (BCP) assisted phase transition		Post deposition with shadow mask effect	Pre-fabricated vertically aligned SID patterns
**Device Parameters and performance**	**Channel Materials**	Exfoliated MoS_2 5~10 layers	**CVD MoS_2 monolayer**	Exfoliated MoS_2 monolayer	Exfoliated MoS_2 monolayer	Exfoliated MoS_2 3 layers	**CVD MoS2 monolayer**	Exfoliated BP few layers	**CVD MoS2 monolayer**
	**Channel length**	~80 nm	8.2 nm	15 nm	4 nm	6*7.5 nm	8*7.5 nm	20 nm	12.6 nm
	**Gate**	60 nm SiN_x (EOT 34 nm)	6 nm HfO_2 (EOT ~2 nm)	10 nm HfO_2 (EOT 3.3 nm)	2.5 nm hBN (EOT ~2.5 nm)	10 nm HfO_2 (EOT 4 nm)	10 nm HfO_2 (EOT 4 nm)	10 nm Al_2O_3 (EOT ~8 nm)	34 nm hBN (EOT ~35 nm)
	**V_g range**	-20~25 V (V_{th} ~-15 V)	-3~2 V (V_{th} ~-1.2 V)	-1.5~1.5 V (V_{th} ~-0.5 V)	-2~2 V (V_{th} ~-0.3 V)	-2~2 V (V_{th} ~-0.7 V)	-3.5~2 V (V_{th} ~-2 V)	4~-4 V P type	-5~16 V (V_{th} ~0.26 V)
	**I_{on} /layer number at V_{ds} = 0.5 V**	~320 μA/μm / (5~10)	~1.3 μA/μm / 1	~60 μA/μm / 1	~9 μA/μm / 1 V_{ds} = 0.1 V	~ 40 μA/μm /3	~ 60 μA/μm / 1	174 μA/μm V_{ds} = 0.1 V	>21.5 μA/μm / 1
	**On/off ratio**	$4*10^5$	$\sim10^6$	$\sim5*10^5$	$\sim10^6$	$\sim10^7$	$3*10^5$	$\sim10^2$	$10^7\sim10^9$
	SS	1200 mV/dec	140 mV/dec	205 mV/dec	208 mV/dec	120 mV/dec	540 mV/dec	~1200 mV/dec	700 mV/dec
**Contact**	**Contact Material**	Graphene top contact	Au bottom contact	Graphene top contact	Graphene bottom contact	1T' phase contact		Au top contact	Au bottom contact
	**R_c**	~0.54 KΩ μm	/	/	3.8 KΩ μm	75 Ω μm		/	~1.2—1.5 KΩ μm
**Feasibility of large-scale and high-density fabrication**		Difficult	Difficult	Difficult	Difficult	Difficult		Possible	**Yes**

Table S2. Comparison between 2DVFETs and Si FinFETs.

	2DVFETs	Intel 14 nm[15]	Intel 10 nm[16]
**Channel materials**	Monolayer 2D semiconductor (eg. MoS_2)	Si fin	Si fin
**Channel length**	12.6 nm	20 nm	18 nm
**Ideal fabrication limit of the length**	1 nm<br>(To overcome the short channel effect, 1 nm gate length is demonstrated[8], and for the spacer, in ideal case, it is also able to be 1 nm to avoid leakage)	?	13.4~16.8 nm[17]
**Channel thickness**	~0.31 nm[18, 19]<br>(Here we didn't consider the van der Waals gap)	8 nm	7 nm
**Gate**	hBN with thickness of 34 nm<br>Single gate	HKMG EOT = 0.9 nm ?<br>Tri-gate	HKMG EOT = 0.9 nm ?<br>Tri-gate
**Simplified schematic illustration**	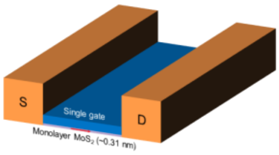	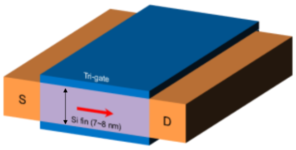	
I_{on}	21.5 μA μm^{-1} at V_{ds} = 0.5 V<br>(at $V_{g.E}$ = ~0.42 V, supposing with the same EOT of 0.9 nm)	0.45 mA μm^{-1} at V_{DD} = 0.5 V<br>1.04 mA μm^{-1} at V_{DD} = 0.7 V	0.76 mA μm^{-1} at V_{DD} = 0.5 V<br>1.78 mA μm^{-1} at V_{DD} = 0.7 V
I_{on} **divided by thickness**	~70 μA μm^{-1} nm^{-1} at V_{ds} = 0.5 V<br>(at $V_{g.E}$ = ~0.42 V, supposing with the same EOT of 0.9 nm)	~56 μA μm^{-1} nm^{-1} at V_{DD} = 0.5 V<br>~130 μA μm^{-1} nm^{-1} at V_{DD} = 0.7 V	~108 μA μm^{-1} nm^{-1} at V_{DD} = 0.5 V<br>~250 μA μm^{-1} nm^{-1} at V_{DD} = 0.7 V
I_{off}	0.2 nA μm^{-1}	10 nA μm^{-1}	10 nA μm^{-1}
**On-off ratio**	10^7~10^9	10^5	<10^6
**Contact**	1.5 kΩ μm	<0.1 kΩ μm	<0.1 kΩ μm
**Sub-threshold swing**	700 mV dec^{-1} → 77 mV dec^{-1}<br>(supposing with the same EOT of 0.9 nm and consider the thermionic limit of 60 mV dec^{-1})[20]	~65 mV dec^{-1}	~70 mV dec^{-1}
**DIBL**	2.5 V/ 0.45 V = 5.5 V V^{-1} → ~147 mV V^{-1}<br>(supposing with the same EOT of 0.9 nm)	~60 mV V^{-1}	~70 mV V^{-1}
**Hysteresis**	Very small	Very small	Very small
**Strategy for critical patterning**	Self-aligned etching or deposition controlled with the spacer thickness	Self-aligned double patterning with 193 nm immersion lithography	Self-aligned quad patterning with 193 nm immersion lithography
**Layout for high density**	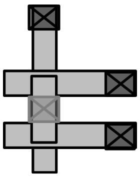	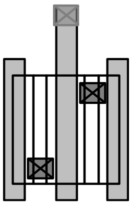	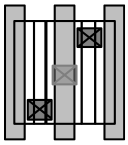

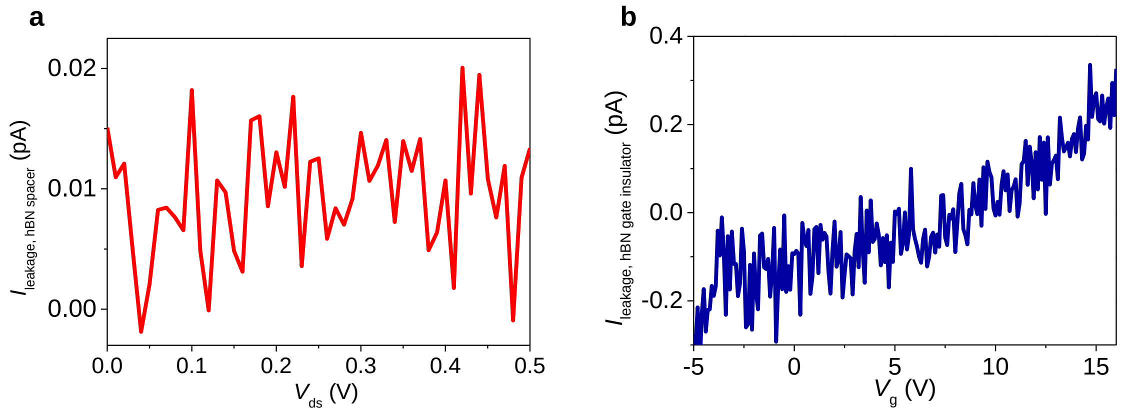

Figure S5. Leakage check for hBN as the insulating spacer and gate insulator. **(a)** Leakage current of the SID pattern with hBN spacer ($I_{leakage,\ hBN\ spacer}$) for the MoS_2-based 2DVFET before MoS_2 channel fabrication. **(b)** Leakage current of the top gate ($I_{leakage,\ hBN\ gate\ insulator}$). The levels of both leakages are below picoampere.

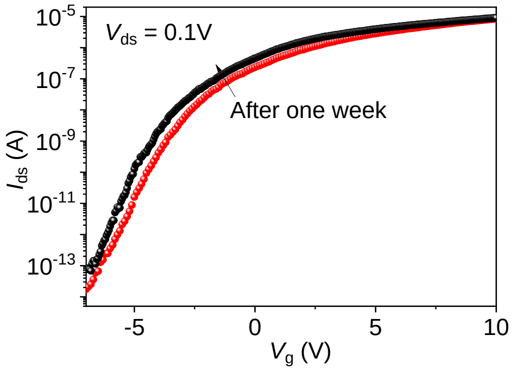

Figure S6. Stability of device performance check for the MoS_2-based 2DVFET. The performance of device is slightly changed (not significantly).

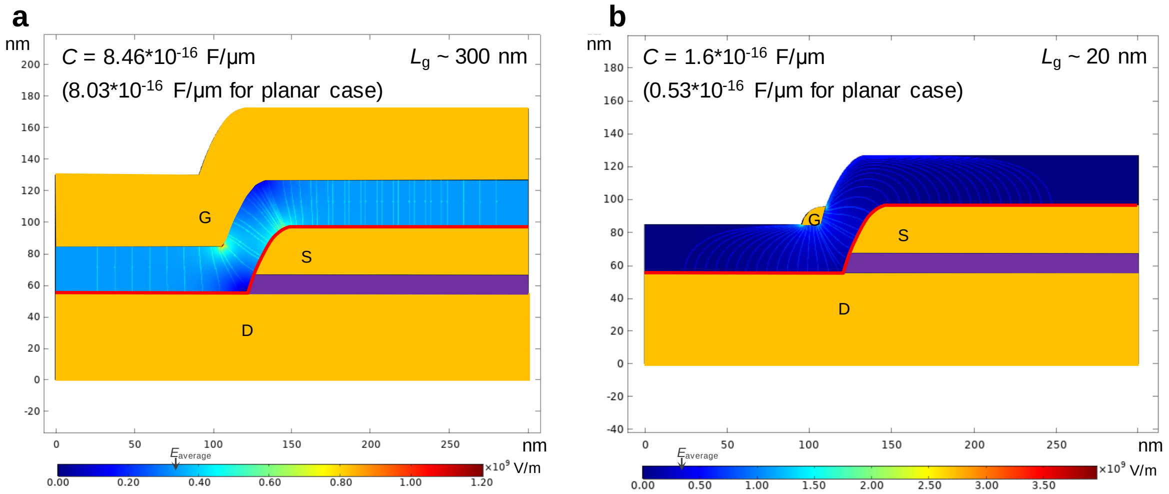

Figure S7. Simulation of the capacitance and the electric field distribution inside the gate dielectric using COMSOL Multiphysics. a) For the simplified structure simulated from the cross-sectional TEM image. b) For the case of reduced gate length. The color bar indicates the electrical field distribution inside the gate dielectric with unit of V/m. The electrodes (source (S), drain (D) and gate (G)), the spacer, and the channel are marked as yellow, purple and red, respectively. A bias of 10 V is applied between the gate electrode and soure/drain with defined equipotential channel to simulate the electric field distribution. In the case of long gate electrode (a), the maximum local electric field at the corner of the gate electrode (~$1.2*10^{9}$ V/m) is ~4 times larger than the average electric field (~$0.33*10^{9}$ V/m). The total capacitance of the simulated structure ($8.46*10^{-16}$ F/μm) is ~5% larger than the value of planar capacitor with the same electrode length ($8.03*10^{-16}$ F/μm). The capacitance is further enhanced when the gate electrode is reduced close to the channel length, with capacitance value ($1.6*10^{-16}$ F/μm) approximately 3 times larger than the value of planar capacitor with the same electrode length ($0.53*10^{-16}$ F/μm). Comparing (a) and (b), it is observed that the capacitance value in 2DVFET is approximately 5 times reduced from $8.46*10^{-16}$ F/μm in the long gate-channel case (300 nm) to $1.6*10^{-16}$ F/μm in the short gate-channel case (20 nm). However, the simulation ratio is much smaller than that of simple length-scale approximation (300 nm/20 nm). It suggests that the locally enhanced electric field at the corner of the gate electrode at the top of the channel will enhance the gate effect to turn on and off the channel more effectively.

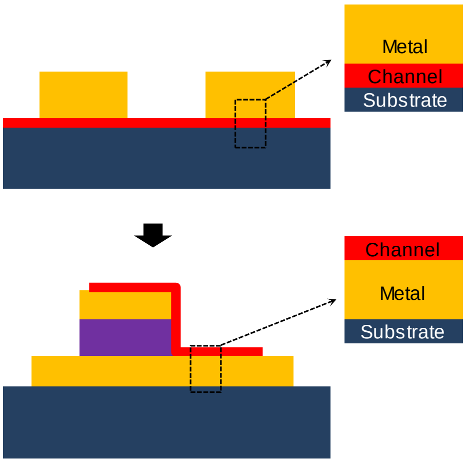

Figure S8. Schematic illustration for comparison between conventional top contact and bottom contact in 2DVFETs. In 2DVFETs, the configuration of the contact is bottom contact, which is different from conventional top-contact FET devices. Bottom contact has been used for some organic semiconductors. However, it is normally not as good as top contact.[21] For 2D materials, the situation is different. A low Schottky barrier has been demonstrated for 2D semiconductors with Au bottom contact after proper annealing.[22]

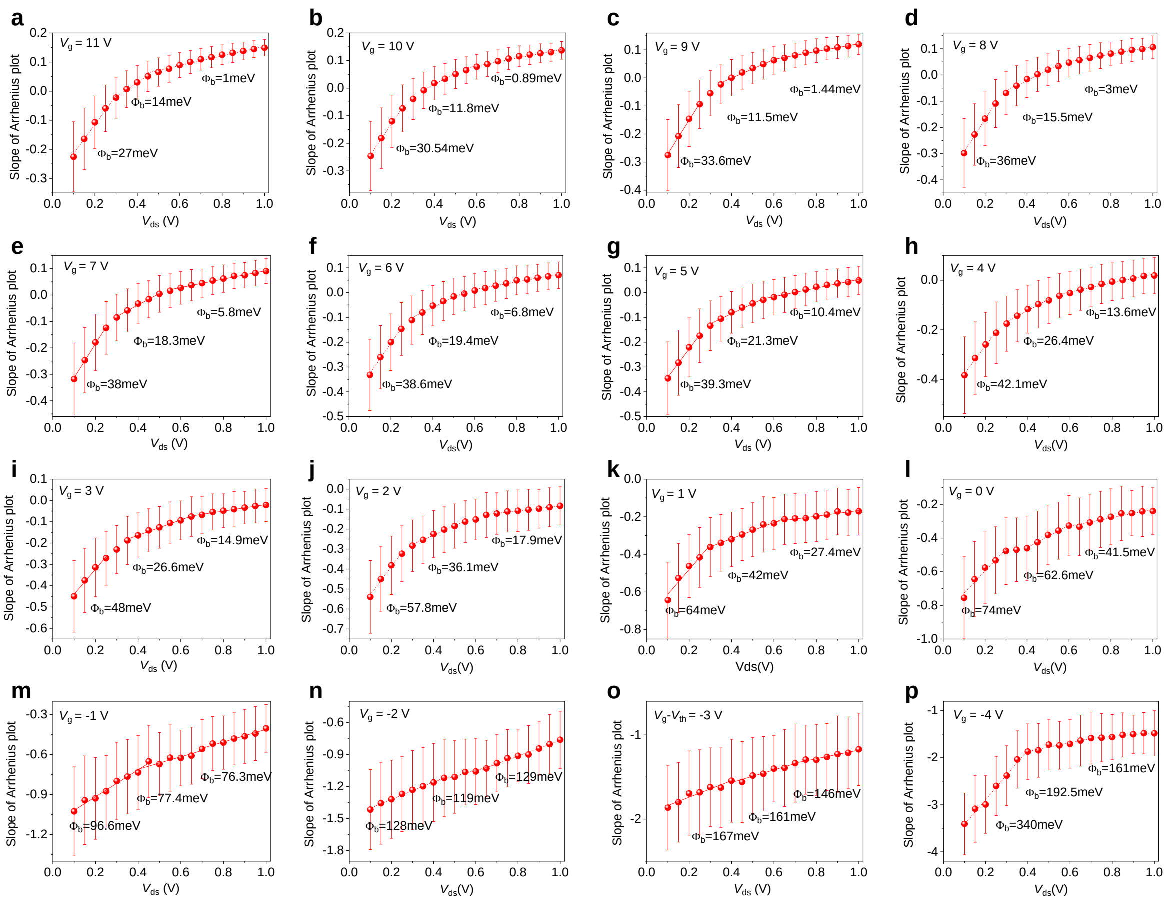

Figure S9. Slopes of the Arrhenius plots as a function of V_{ds} for the CVD MoS_2-based 2DVFET for gate biases from 11 V to -4 V. As the curves are not linear, we estimate three separated near-linear ranges (0.1–0.3 V, 0.3–0.6 V, and 0.6–1.0 V) to extract the y-intercepts for SBH calculation.

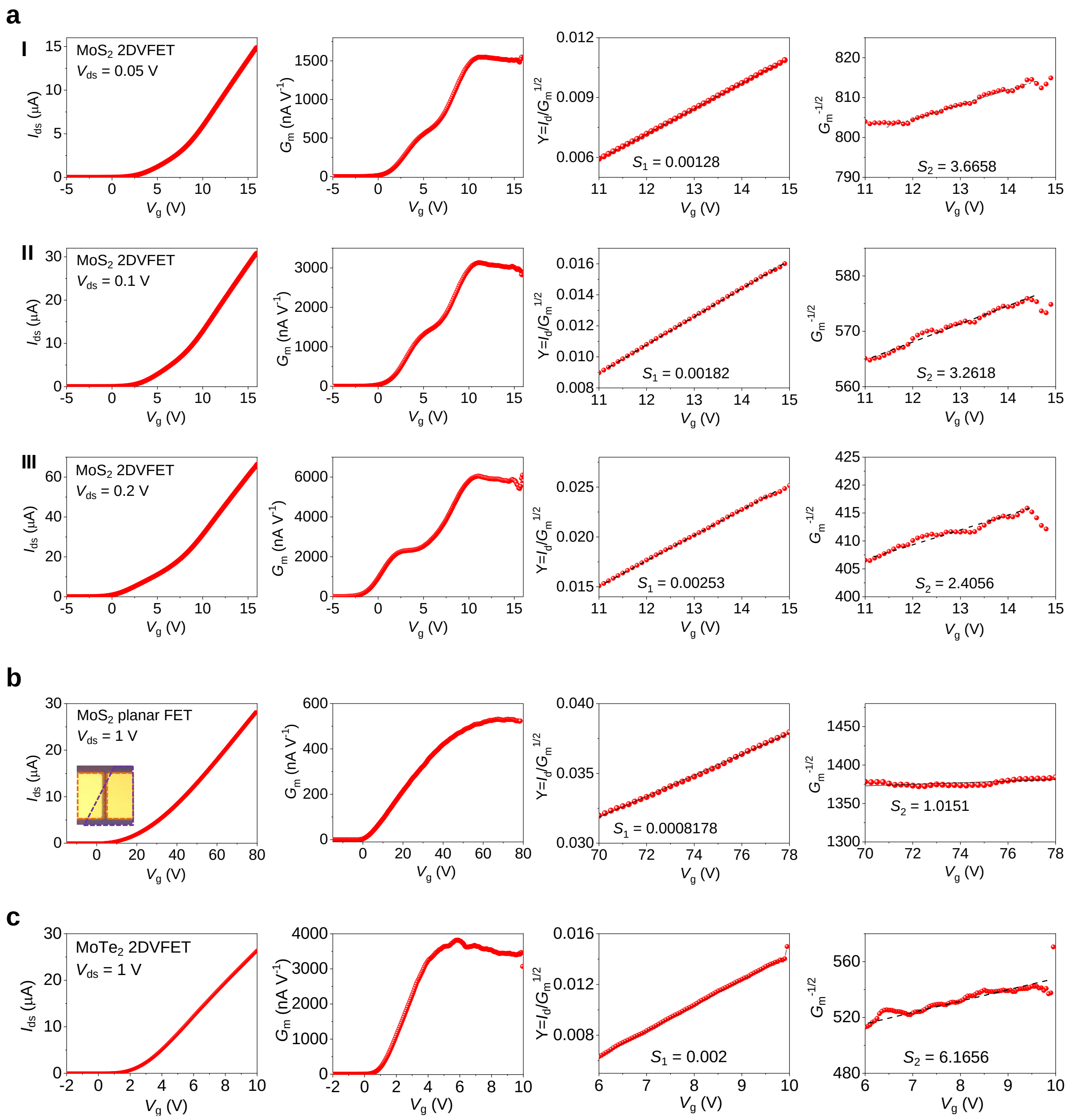

Figure S10. Detailed processes of the Y function method for contact resistance calculation.[23, 24] **(a)** (I–III), MoS_2-based 2DVFET at V_{ds} of 0.05, 0.1, and 0.2 V. **(b)** MoS_2 planar FET at V_{ds} = 1 V. **(c)** $MoTe_2$-based 2DVFET at V_{ds} = 1 V. Here, $G_m = dI_{ds}/dV_g$, $Y = I_{ds}/G_m^{-1/2}$, S_1 is the slope of Y–V_g curves, and S_2 is the slope of $G_m^{-1/2}$ –V_g curves. The contact resistance R_c can be calculated as $R_c = V_{ds}*S_1^{-1}*S_2 *W$, where W is the width of the contact. For MoS_2-based 2DVFET, R_c is calculated as 1.16, 1.45, and 1.54 kΩ μm at V_{ds} of 0.05, 0.1, and 0.2 V, respectively. For MoS_2 planar FET, R_c is approximately 31 kΩ μm. For $MoTe_2$-based 2DVFET, R_c is approximately 1.85 kΩ μm.

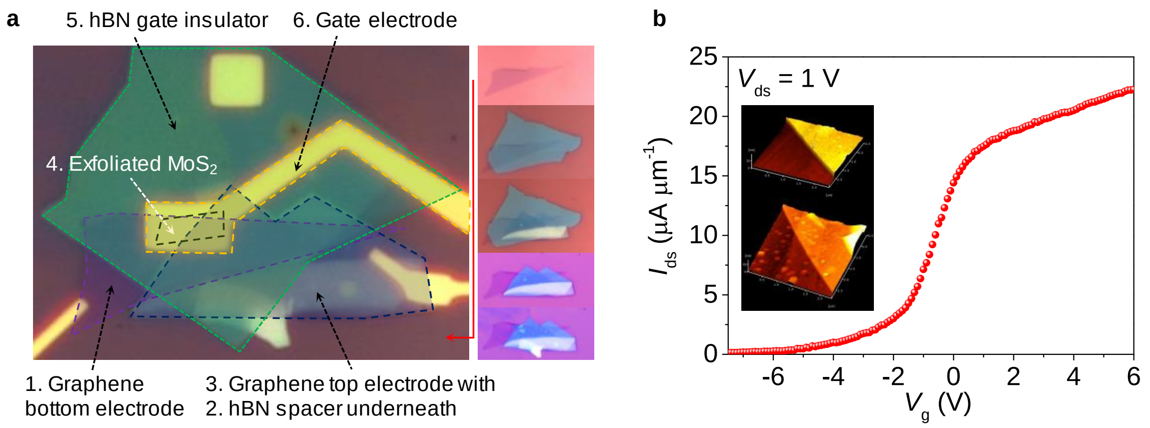

Figure S11. Device using graphene as the contact electrodes for 2DVFETs. **(a)** Optical images of the final fabricated device with six component layers (left) and key fabrication steps (right). The fabrication processes are almost similar to the one described in the main text. Graphene is used for both bottom and top electrodes instead of metal. The top graphene layer could be used as a mask for selectively etching hBN with SF_6 plasma. After SF_6 plasma treatment, the graphene-hBN-graphene SID pattern was annealed in high vacuum at 150 °C before transferring MoS_2. **(b)** Transfer curve of the device and AFM images of the graphene-hBN-graphene SID pattern before and after MoS_2 transfer (inset). The thickness of the hBN spacer and the channel width in this device are 16 nm and 1.5 μm, respectively. The on-current density at V_{ds} = 1 V and V_g = 6 V is approximately 22 μA μm^{-1}.

Table S3. Summary of main issues to realize ideal 2DVFETs.

	Key issues for ideal 2DVFETs
**Channel**	**1)** To avoid short-channel effect, thickness of the channel should be small enough. And this is the key advantage of atomically thin 2D semiconductors as channel materials of FETs.[8]<br>**2)** In ideal case, if the channel length can shrink to the mean free path of the carriers, scattering in the channel will be minimal, and the carrier transport will approach the ballistic transport. The on current will be significantly improved. The Fermi function, carrier velocity, transmission probability and density of energy states turn to be the key considered material-related parameters for channel materials.[25]<br>**3)** Wafer scale fabrication requires wafer scale channel materials. For 2D semiconductors, wafer scale growth and transfer is becoming possible.[26-29]<br>**4)** Channel material should attach closely with the SID patterns especially at the step part to realize the short channel length defined by the spacer thickness. The flexibility of 2D semiconductors[30] is another advantage in the view of fabrication. Reducing the SID pattern height and post-annealing after etching the 2D channel to defined size will enhance the attachment.
**Insulating spacer**	**1)** The spacer layer should also be thin and flat to realize the short channel length. Layered hBN[28] is a promising candidate, and nanometer-thin oxides deposited with ALD[31] are other good candidates available for industry.<br>**2)** Leakage current should be small enough, and it requires high quality of insulators with proper thickness.<br>**3)** Wafer scale fabrication also requires wafer scale materials of insulating spacer. Wafer scale growth of hBN[28] and ALD deposition of oxides[31] is available.<br>**4)** Roughness issue.<br>The edge roughness of the insulating spacer will induce the roughness of the channel. In our experiments, the roughness of the hBN edge is mainly determined by the etching process (reactive ion etching with SF_6 in the present work). Such a rough edge of hBN can reduce the mobility of our devices. A feasible solution for improving the roughness of the channel is to generate a quasi-suspended 2D semiconductor vertical channel as described in the following figure. The hBN underneath the top electrode can be selectively etched further in the lateral direction to form a partial empty spacer, and the suspended vertical channel can be generated after transferring 2D semiconductors. It is noted that a similar approach has been demonstrated in Nature nanotechnology 2019, 14, 579. [32]<br>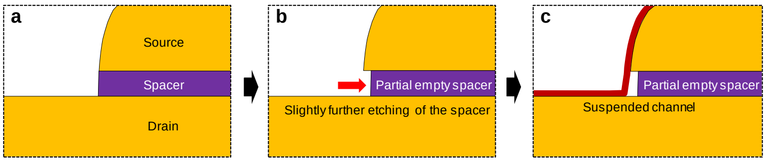<br><br>Schematic of feasible processes to generate a quasi-suspended 2D semiconductor vertical channel. a) Original source-insulating spacer-drain pattern. b) Further etching the spacer underneath the top electrode. c) Suspended channel after transferring 2D semiconductors.
**Contact electrode**	**1)** Ohmic or near Ohmic contact is required to realize high on current density with small contact resistance. Metallic materials with work function matched with 2D channel materials is required. Van der Waals type contact between metal and 2D semiconductors in 2DVFETs will help to avoid the pinning effect and realize such matching.[33, 34] Fabrication in glove box will be one resolution to avoid the surface oxidization of metals with low work function. Graphene[35] or other metallic layered materials[36] would also be candidates as contact materials.<br>**2)** The contact electrode should also be thin and flat to help maintain the attachment between the channel material and the SID pattern. Ultra-thin metallic electrodes would be possible with layered metallic materials[35, 36] or normal metals using ALD deposition[37].

Table S3 continued. Summary of main issues to realize ideal 2DVFETs.

Gate

1) Gate insulator should be thin with small EOT to realize small *SS* values for the device. High-K metal gate is already well applied in industry[38], and it is also available to fabricate on 2D semiconductors[39].

2) Gate leakage current should also be small enough.

3) Gate length and overlap capacitance issue.

Gate length is as important as the channel length in high-performance and low-power logic transistors. Only scaling down the channel length while not gate length will induce large overlap between the gate and source/drain electrodes, inducing the parasitic capacitance. Such capacitance will enlarge the total capacitance and affect both the intrinsic gate delay (CV/I) and energy-delay product ($CV/I \times CV^2$) of the transistor.[17, 40] However, in the current work, we mainly focused on the concept demonstration for the new device structure design of vertical-type short channel transistors, while the optimization of gate length is not investigated.

Here we would like to propose one feasible solution to scale down the gate length in our 2DVFETs. As shown in the following figure, with the step-shape feature of 2DVFETs, we can take use of the sidewall etching technique normally used in the spacer patterning[15, 41] in industry, and etch back the gate electrode to decrease the gate length, approaching the size of channel length.

As discussed above, the long gate length can strongly affect the speed of the devices. To estimate the speed limit of the device, we consider the intrinsic gate delay defined as $\tau = CV/I \sim C/G_m$, where C is the total capacitance including the overlap capacitance, and G_m is the transconductance defined as $G_m = \mathrm{d}I_{ds}/\mathrm{d}V_g$. For simplified estimation, we consider that the total capacitance contains mainly two parts: gate-channel capacitance ($C_{gc} = c \times L_{channel} \times W$) and overlap capacitance ($C_{ov} = c \times (L_{gate} - L_{channel}) \times W$), where c is the capacitance density assuming to be same for both C_{gc} and C_{ov}. We can estimate $\tau = [c \times W \times L_{channel} \times (L_{gate}/ L_{channel})]/G_m$. In our device with MoS_2, $G_m \sim 2.2\ \mu A\ \mu m^{-1}\ V^{-1}$ ($V_{ds} = 0.5$ V), $c \sim 100$ nF cm^{-2} for the 34-nm thick hBN gate insulator, $L_{channel}$ ~12.6 nm and $W \sim 8.1\ \mu m$, $\tau \sim 5\ ps \times (L_{gate}/ L_{channel})$ at $V_{ds} = 0.5$ V. For $MoTe_2$ based device, $\tau \sim 1.5\ ps \times (L_{gate}/L_{channel})$ at $V_{ds} = 2$ V. Supposing that the gate length was reduced approaching the size of channel length, the limit turns to be at the level of 10^0~10^1 ps. It is over one order larger than the ultimate intrinsic gate delay for Si-FETs (~0.1 ps).[42] This value should be improved by enhancing the transconductance, which is mainly limited by the contact resistance.

Taking consideration of the local enhancement effect as discussed in Figure S7, we can see that the capacitance value in our 2DVFET is approximately 5 times reduced from 8.46×10^{-16} F/μm in the long gate length case (300 nm) to 1.6×10^{-16} F/μm in the short gate length case (20 nm). The difference is expected from the parasitic capacitance. However, the simulation ratio is much smaller than that of expected simple length-scale approximation (300 nm/20 nm). The origin of this phenomenon is caused by the local field enhancement. It suggests that the local field enhancements could improve the effective gate-channel capacitance.

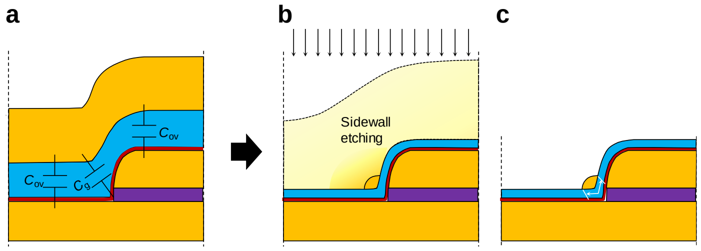

a) Simplified schematic illustration to show the gate length and overlap capacitance issue. b, c) Schematic illustration of sidewall etching to reduce the gate length. The basic idea is to take advantage of the step-shape feature of 2DVFETs to post-etch the deposited electrode.

References

[1] M. Chen, C. Lin, L. Kai-Hsin, L. Li, C. Chen, C. Cheng-Hao, L. Ming-Dao, Y. Chen, Y. Hou, C. Lin, C. Chen, B. Wu, C. Wu, I. Yang, Y. Lee, Y. Wen-Kuan, T. Wang, F. Yang, C. Hu, *IEDM Tech. Dig.* **2014**, 33.5.1–33.5.4, DOI: 10.1109/IEDM.2014.7047163.

[2] M. Chen, K. Li, L. Li, A. Lu, M. Li, Y. Chang, C. Lin, Y. Chen, Y. Hou, C. Chen, B. Wu, C. Wu, I. Yang, Y. Lee, J. Shieh, W. Yeh, J. Shih, P. Su, A. B. Sachid, T. Wang, F. Yang, C. Hu, *IEDM Tech. Dig.* **2015**, 32.2.1–32.2.4, DOI: 10.1109/IEDM.2015.7409813.

[3] K.-S. Li, B.-W. Wu, L.-J. Li, M.-Y. Li, C.-C. K. Cheng, C.-L. Hsu, C.-H. Lin, Y.-J. Chen, C.-C. Chen, C.-T. Wu, *Symp. VLSI Tech. Dig.* **2016**, DOI: 10.1109/VLSIT.2016.7573375.

[4] L. Yang, R. T. P. Lee, S. S. P. Rao, W. Tsai, P. D. Ye, *DRC Tech. Dig.* **2015**, 237–238, DOI: 10.1109/DRC.2015.7175655.

[5] C. D. English, K. K. Smithe, R. L. Xu, E. Pop, *Int. El. Devices Meet* **2016**, 5, 1.

[6] W. Cao, W. Liu, J. Kang, K. Banerjee, *IEEE Electron Device Lett.* **2016**, 37, 1497.

[7] Z. Yang, X. Liu, X. Zou, J. Wang, C. Ma, C. Jiang, J. C. Ho, C. Pan, X. Xiao, J. Xiong, *Adv. Funct. Mater.* **2017**, 27.

[8] S. B. Desai, S. R. Madhvapathy, A. B. Sachid, J. P. Llinas, Q. Wang, G. H. Ahn, G. Pitner, M. J. Kim, J. Bokor, C. Hu, H.-S. P. Wong, A. Javey, *Science* **2016**, 354, 99.

[9] Y. Liu, J. Guo, Y. Wu, E. Zhu, N. O. Weiss, Q. He, H. Wu, H.-C. Cheng, Y. Xu, I. Shakir, *Nano lett.* **2016**, 16, 6337.

[10] K. Xu, D. Chen, F. Yang, Z. Wang, L. Yin, F. Wang, R. Cheng, K. Liu, J. Xiong, Q. Liu, *Nano lett.* **2017**, 17, 1065.

[11] A. Nourbakhsh, A. Zubair, S. Huang, X. Ling, M. S. Dresselhaus, J. Kong, S. D. Gendt, T. Palacios, *Symp. VLSI Tech. Dig.* **2015**, T28–T29, DOI: 10.1109/VLSIT.2015.7223690.

[12] L. Xie, M. Liao, S. Wang, H. Yu, L. Du, J. Tang, J. Zhao, J. Zhang, P. Chen, X. Lu, *Adv. Mater.* **2017**, 29.

[13] A. Nourbakhsh, A. Zubair, R. N. Sajjad, A. Tavakkoli KG, W. Chen, S. Fang, X. Ling, J. Kong, M. S. Dresselhaus, E. Kaxiras, *Nano lett.* **2016**, 16, 7798.

[14] J. Miao, S. Zhang, L. Cai, M. Scherr, C. Wang, *ACS nano* **2015**, 9, 9236.

[15] S. Natarajan, M. Agostinelli, S. Akbar, M. Bost, A. Bowonder, V. Chikarmane, S.

Chouksey, A. Dasgupta, K. Fischer, Q. Fu, T. Ghani, M. Giles, S. Govindaraju, R. Grover, W. Han, D. Hanken, E. Haralson, M. Haran, M. Heckscher, R. Heussner, P. Jain, R. James, R. Jhaveri, I. Jin, H. Kam, E. Karl, C. Kenyon, M. Liu, Y. Luo, R. Mehandru, S. Morarka, L. Neiberg, P. Packan, A. Paliwal, C. Parker, P. Patel, R. Patel, C. Pelto, L. Pipes, P. Plekhanov, M. Prince, S. Rajamani, J. Sandford, B. Sell, S. Sivakumar, P. Smith, B. Song, K. Tone, T. Troeger, J. Wiedemer, M. Yang, K. Zhang, *IEDM Tech. Dig.* **2014**, 3.7.1–3.7.3, DOI: 10.1109/IEDM.2014.7046976.

[16] C. Auth, A. Aliyarukunju, M. Asoro, D. Bergstrom, V. Bhagwat, J. Birdsall, N. Bisnik, M. Buehler, V. Chikarmane, G. Ding, Q. Fu, H. Gomez, W. Han, D. Ha nken, M. Haran, M. Hattendorf, R. Heussner, H. Hiramatsu, B. Ho, S. Jaloviar, I. Jin, S. Joshi, S. Kirby, S. Kosaraju, H. Kothari, G. Leatherman, K. Lee, J. Le ib, A. Madhavan, K. Marla, H. Meyer, T. Mule, C. Parker, S. Parthasarathy, C. Pelto, L. Pipes, I. Post, M. Prince, A. Rahman, S. Rajamani, A. Saha, J. D. Sa ntos, M. Sharma, V. Sharma, J. Shin, P. Sinha, P. Smith, M. Sprinkle, A. S. A mour, C. Staus, R. Suri, D. Towner, A. Tripathi, A. Tura, C. Ward, A. Yeoh, *IEDM Tech. Dig.* **2017**, 29.1.1–29.1.4, DOI: 10.1109/IEDM.2017.8268472.

[17] P. Young, *J. Phys. D: Appl. Phys.* **1968**, 1, 936.

[18] X. Cui, Z. Kong, E. Gao, D. Huang, Y. Hao, H. Shen, C.-a. Di, Z. Xu, J. Zheng, D. Zhu, *Nat. commun.* **2018**, 9, 1301.

[19] C. Qiu, Z. Zhang, M. Xiao, Y. Yang, D. Zhong, L.-M. Peng, *Science* **2017**, 355, 271.

[20] A. M. Ionescu, H. Riel, *Nature* **2011**, 479, 329.

[21] P. Cosseddu, A. Bonfiglio, *Thin Solid Films* **2007**, 515, 7551.

[22] D. Qi, Q. Wang, C. Han, J. Jiang, Y. Zheng, W. Chen, W. Zhang, A. T. S. Wee, *2D Mater.* **2017**, 4, 045016.

[23] H.-Y. Chang, W. Zhu, D. Akinwande, *Appl. Phys. Lett.* **2014**, 104, 113504.

[24] C. Liu, Y. Xu, Y.-Y. Noh, *Mater. Today* **2015**, 18, 79.

[25] A. D. Franklin, *Science* **2015**, 349, aab2750.

[26] K.-K. Liu, W. Zhang, Y.-H. Lee, Y.-C. Lin, M.-T. Chang, C.-Y. Su, C.-S. Chang, H. Li, Y. Shi, H. Zhang, *Nano lett.* **2012**, 12, 1538.

[27] H. Yu, M. Liao, W. Zhao, G. Liu, X. Zhou, Z. Wei, X. Xu, K. Liu, Z. Hu, K. Deng, *ACS*

nano **2017**, 11, 12001.

[28] J. S. Lee, S. H. Choi, S. J. Yun, Y. I. Kim, S. Boandoh, J.-H. Park, B. G. Shin, H. Ko, S. H. Lee, Y.-M. Kim, Y. H. Lee, K. K. Kim, S. M. Kim, *Science* **2018**, 362, 817.

[29] K. Kang, K.-H. Lee, Y. Han, H. Gao, S. Xie, D. A. Muller, J. Park, *Nature* **2017**, 550, 229.

[30] D. Akinwande, C. J. Brennan, J. S. Bunch, P. Egberts, J. R. Felts, H. Gao, R. Huang, J.-S. Kim, T. Li, Y. Li, *Extreme Mech. Lett.* **2017**, 13, 42.

[31] R. W. Johnson, A. Hultqvist, S. F. Bent, *Mater. Today* **2014**, 17, 236.

[32] J. Lenz, F. del Giudice, F. R. Geisenhof, F. Winterer, R. T. Weitz, *Nat. Nanotechnol.* **2019**, 14, 579.

[33] Y. Liu, J. Guo, E. Zhu, L. Liao, S.-J. Lee, M. Ding, I. Shakir, V. Gambin, Y. Huang, X. Duan, *Nature* **2018**, 557, 696.

[34] Y. Wang, J. C. Kim, R. J. Wu, J. Martinez, X. Song, J. Yang, F. Zhao, A. Mkhoyan, H. Y. Jeong, M. Chhowalla, *Nature* **2019**, 568, 70.

[35] L. Yu, Y.-H. Lee, X. Ling, E. J. Santos, Y. C. Shin, Y. Lin, M. Dubey, E. Kaxiras, J. Kong, H. Wang, *Nano lett.* **2014**, 14, 3055.

[36] S. Cho, S. Kim, J. H. Kim, J. Zhao, J. Seok, D. H. Keum, J. Baik, D.-H. Choe, K. J. Chang, K. Suenaga, S. W. Kim, Y. H. Lee, H. Yang, *Science* **2015**, 349, 625.

[37] H. Kim, *J. Vac. Sci. Technol. B* **2003**, 21, 2231.

[38] K. Mistry, C. Allen, C. Auth, B. Beattie, D. Bergstrom, M. Bost, M. Brazier, M. Buehler, A. Cappellani, R. Chau, C. Choi, G. Ding, K. Fischer, T. Ghani, R. Grover, W. Han, D. Hanken, M. Hattendorf, J. He, J. Hicks, R. Huessner, D. Ingerly, P. Jain, R. James, L. Jong, S. Joshi, C. Kenyon, K. Kuhn, K. Lee, H. Liu, J. Maiz, B. McIntyre, P. Moon, J. Neirynck, S. Pae, C. Parker, D. Parsons, C. Prasad, L. Pipes, M. Prince, P. Ranade, T. Reynolds, J. Sandford, L. Shifren, J. Sebastian, J. Seiple, D. Simon, S. Sivakumar, P. Smith, C. Thomas, T. Troeger, P. Vandervoorn, S. Williams, K. Zawadzki, *IEDM Tech. Dig.* **2007**, 247–250, DOI: 10.1109/IEDM.2007.4418914.

[39] X. Zou, J. Wang, C. H. Chiu, Y. Wu, X. Xiao, C. Jiang, W. W. Wu, L. Mai, T. Chen, J. Li, *Adv. Mater.* **2014**, 26, 6255.

[40] R. Chau, S. Datta, M. Doczy, B. Doyle, B. Jin, J. Kavalieros, A. Majumdar, M. Metz, M. Radosavljevic, *IEEE T. Nanotechnol.* **2005**, 4, 153.

[41] C. Yang-Kyu, K. Tsu-Jae, H. Chenming, *IEEE Trans. Electron Devices* 2002, 49, 436.

[42] www.itrs2.net/2013-itrs.html
